# Application of plastic conjugated materials in the repair of sports injury

**DOI:** 10.3389/fchem.2023.1273726

**Published:** 2023-10-02

**Authors:** Peng Li, Jihe Zhou

**Affiliations:** ^1^ Graduate School of Chengdu Sports University, Chengdu Sports University, Chengdu, China; ^2^ School of Physical Education and Health, Zunyi Medical University, Zunyi, China

**Keywords:** conjugated material, sports injury, degradability of conjugated materials, compressive strength, compression modulus, proportion of polylactide content

## Abstract

For professional athletes or fitness crowd who often participate in sports, sports injury are more common. However, the traditional repair materials for sports injury have many problems, such as long recovery period and poor repair effect. In recent years, many studies have found that conjugated materials have good stability, small side effects and other excellent characteristics, and conjugated materials are used in sports injury repair materials. In order to study the repair effect of conjugated materials on sports injury tissues, this paper prepared nitrogen porphyrin conjugated organic skeleton materials with pyrrole and p-benzaldehyde as substrates, used chemical synthesis and selective laser sintering technology to form plastic conjugated scaffold materials with polyvinyl alcohol, polylactide and conjugated materials, and established mechanical properties and constitutive formula to evaluate the performance characteristics of repair materials. In order to test the effect of plastic conjugated materials on sports injury, experiments were carried out from the aspects of degradability, mechanical properties of repair materials, repair effect of radius defect and new bone formation area. Experimental data: The proportion of newly formed bone area at weeks 3, 6, and 9 using this method was 32%, 52%, and 68%, while the proportion of newly formed bone area at weeks 3, 6, and 9 using traditional methods was 12%, 18%, and 23%, indicating that this method had better bone repair effects than traditional methods. The research in this paper provided a new idea for the application of plastic conjugated materials in the field of sports medicine.

## 1 Introduction

In the context of national fitness, more and more people participate in sports, and the projects they participate in also have diversified characteristics, among which there are some sports projects with fierce confrontation and high difficulty technology, and the incidence of sports injury caused by this is also high. However, the traditional repair methods for sports injury have the problems of low targeting and slow curative effect. The use of plastic conjugated materials can effectively solve these problems, which also promotes the urgent need for plastic conjugated materials in the field of sports medicine. With the in-depth study of the role of conjugated materials in repairing sports injury, plastic conjugated materials have shown great advantages over other materials in treating sports injury.

With the increase of the incidence of sports injury, the repair of sports injury has become a research hotspot in the field of sports science, showing a broad research prospect. Qi Hao Looi pointed out that more and more people were participating in sports, and the number of sports related injuries was gradually rising. Timely and effective treatment was particularly important for sports injury. Because of the sports injury that was difficult to treat with traditional therapy, he conducted research on the treatment of sports injury by mesenchymal stem cells. Mesenchymal stem cells secreted paracrine signaling factors to regulate host immune response, promote angiogenesis, enhance cell migration and survival, and prevent fibrosis. The experiment showed that mesenchymal stem-cell therapy was safe and effective in treating sports injury such as muscles, ligaments, tendon bones, cartilage and nervous tissue, and was applicable to various sports injury (Qi et al., 2020). Michael G. Azzam pointed out that improper preparation before exercise was likely to lead to various sports injury, one of which was rotator cuff tear. Especially in the youth population, if rotator cuff tear injuries were not identified in a timely manner, the condition would become increasingly severe. The scholar found that all patients who participated in at least one sport suffered from sports injury of varying degrees by searching past case records. Among them, 2 cases were complete tendon tears, 5 cases were tendon bone avulsion injuries, and 18 cases were advanced partial tears. 14 patients (56%) received single row repair for rotator cuff tears, and 11 patients (44%) received double row repair. The results indicated that in patients with timely treatment of rotator cuff tears, surgical repair of high-grade partial thickness and complete rotator cuff tears achieved success (Michael et al., 2018). Alexa G. Gagliardi pointed out that the common sports injury of sports personnel was anterior cruciate ligament strain. However, the failure rate of anterior cruciate ligament repair for patients using suture ligament reinforcement in traditional methods was still high. To address this issue, the scholar conducted a study on anterior cruciate ligament repair. He compared two methods of anterior cruciate ligament repair by suture ligament enhancement and anterior cruciate ligament reconstruction by autologous quadriceps tendon-patella transplantation, setting up two sets of experiments with a population of adolescent patients (7–18 years old). The results showed that compared with the autologous quadriceps tendon-patella transplantation, the risk of failure of suture ligament enhancement was significantly increased (Alexa et al., 2019). Ahmed Khalil Attia research mentioned that the traditional treatment for acute achilles tendon rupture was open surgical repair. In order to improve the repair function, he proposed minimally invasive procedure repair. He set up a randomized controlled trial meeting the conditions of meta-analysis, with a total of 522 patients. 260 patients received open repair and 262 received minimally invasive procedure. The results showed that the recurrence rate of open surgery was 2.5% and the recurrence rate of minimally invasive procedure was 1.5%. The average operation time of open repair and minimally invasive repair was 51 min and 29.7 min, respectively. The average total superficial infection rate of open repair and minimally invasive repair was 6.0% and 0.4%, respectively. Research found that minimally invasive repair was a safe and reliable technology (Ahmed et al., 2023). At present, sports injury repair methods have achieved good results in clinical experiments. However, with the development of medicine, people have an urgent need to reduce the repair period of sports injury and improve the effect of injury repair. Current sports injury repair methods cannot solve these problems.

Sports injury has a very serious negative impact on the health of sports participants, and may even lead to the loss of health and sports potential. Traditional sports injury repair materials can not meet the current demand, people began to explore the relationship between plastic conjugated materials and sports injury. Zeeshan Sheikh pointed out that sports bone tissue injury was always a research hotspot in the field of sports medicine. Aluminum silicate was a good choice of biomaterials for bone grafting, but due to the lack of bone induction, some plastic compounds were combined in Aluminum silicate matrix to form plastic conjugate materials, thus promoting bone regeneration. The experimental subjects were 24 bilateral bone defect rats with mandibular defects. The control group used Aluminum silicate as the damage repair material, while the experimental group used plastic conjugated material as the repair material. The study found that the bone formation rate of the plastic conjugated material was higher than that of the control group in the second and fourth weeks. This research provided a promising alternative to bone grafting for bone regeneration and repair in orthopaedic applications (Zeeshan et al., 2020). Mduduzi N. Sithole suggested that progressive methods were needed in bone tissue engineering to repair and regenerate bone defects caused by trauma or disease. In his research, sodium alginate was used as a biological ink, which interacted with polyethylenimine solution on biological printing, formed polyelectrolyte complex through ionic bond, and designed a plastic conjugate material scaffold *in situ*. The porosity and pore size of the scaffold material were 60% and 360µ m respectively, and the biomechanical evaluation showed a Young’s modulus of 18.37 MPa (Mega pascal). The research results highlighted that the current form of plastic conjugated polymer scaffold material had the mechanical ability to be used for some bone tissue engineering applications, and pointed out that the plastic conjugated material scaffold had the potential to be used as a repair engineering scaffold for bone tissue sports injury (Mduduzi et al., 2018). Songhao Luo pointed out that conjugated microporous polymers were a new kind of materials that could be used in artificial photosynthetic systems. He described the light trapping ability and energy transfer phenomenon in the supermolecule structure of conjugated microporous polymers, which had good catalytic performance. He added conjugated microporous polymers to the synthesis of sports injury repair materials, and found that under the catalytic action of conjugated microporous polymers, the reaction rate of sports injury repair materials was accelerated. Meanwhile, this study provided guidance for the rational design of polymers with excellent catalytic performance, and also indicated that conjugated microporous polymers could be applied to sports damage (Songhao et al., 2020). Baker Matthew A pointed out that in the past 15 years, controllable synthesis of π-conjugated polymers was discovered, and this was one of the important components of sports bone injury repair materials. Aromatic building block chain growth polymerization (known as catalyst transfer polycondensation) became a powerful method for controlling the synthesis of conjugated polymers. The plastic conjugated polymer could form a skeleton material with predictable molecular weight and relatively narrow molar mass distribution and customization. This plastic conjugated skeleton material had higher activity, stability, and mechanical properties, which could effectively promote the repair of sports induced bone damage (Baker et al., 2018). It can be seen that these studies have conducted detailed studies on plastic conjugated materials, providing valuable information for this article.

Sports are popular with the public in recent years. With the diversity of sports types, there are more and more types of sports injury (Gatenby, 2021). The repair time, repair methods and repair effects of sports injury have become a hot topic. This article used plastic conjugated materials as repair materials for sports bone injuries, and analyzed the utility of plastic conjugated materials from the perspectives of degradation performance, compressive strength, compressive modulus, cumulative pore volume, and porosity.

## 2 Application of plastic conjugated materials in the repair of sports injury

### 2.1 Evaluation of sports participation and incidence of sports injury

With the improvement of living standards, the public is paying more and more attention to physical health, which has also sparked a wave of nationwide participation in sports. Exercise can not only promote physical fitness, strengthen muscles and bones, but also enhance heart and lung function, enhance immunity, and cultivate a healthy and positive lifestyle without actively participating in sports. This article analyzes the importance that the public places on sports, and calculates the proportion of people participating in sports among different age groups in the past 3 years. According to Table 1, the proportion of people participating in sports between the ages of 7 and 18 is the largest among all age groups. Teenagers aged 7–18 are in physical development, and the majority of the population in this age group are students. They also spend more time participating in sports than other age groups; the proportion of people aged 19 to 29 who participate in sports is the second, and this age group mostly participates in physical exercise due to personal hobbies; The proportion of people in other age groups participating in sports is lower than those in the first two age groups, but the number of people participating in sports every year is also gradually increasing. In summary, people’s attention to sports is gradually increasing.

**TABLE 1:** Proportion of people of different age groups participating in sports in the past 3 years.

Age group	2021 (%)	2022 (%)	2023 (%)
7–18 years old	65.8	75.8	82.3
19–29 years old	61.2	71.8	79.6
30–39 years old	59.5	63.9	68.3
40–49 years old	53.2	56.7	61.6
50–59 years old	51.2	53.0	57.2
60–69 years old	46.8	49.4	52.2
70–79 years old	39.5	41.1	43.3

**FIGURE 1:**
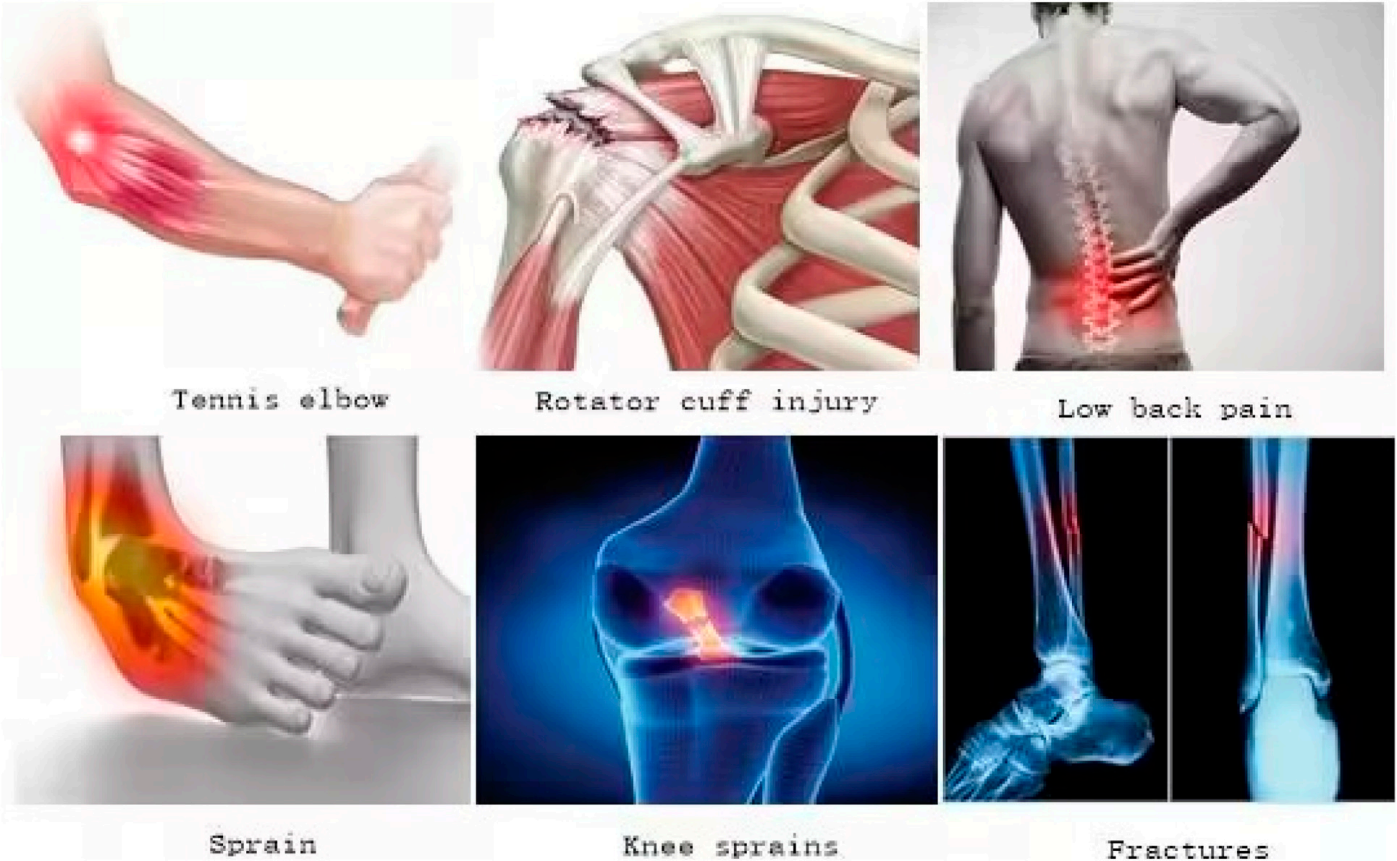
Picture of sports injury.

However, exercise is also a double-edged sword. When people exercise in an unscientific way or encounter an accident, they would suffer from collision, sprain, fall, repetitive strain injury and other sports injury (Caroline et al., 2019; Torbjorm et al., 2019). In order to better understand the relevant knowledge of sports injury, this paper collected some common injury pictures in the process of sports, as shown in Figure 1.

Whether it is the general public or professional athletes, sports injuries always occur during physical exercise (Rasmus et al., 2019; Nian, 2022). In order to understand the occurrence of sports injury among the public, this paper has made statistics on the incidence of male and female sports injury, as shown in Table 2. From the total incidence of sports injury, the incidence of wrist injury, rotator cuff sprain and ankle sprain is relatively high, which are 56.8%, 46.5% and 44.6% respectively, of which the incidence of wrist injury has exceeded 50%; from a longitudinal perspective, the incidence of wrist injury is the highest in males at 30.2%, and the highest and highest in females at 26.6%. From the perspective of the recurrence rate of sports injury, the value of each index is not much lower than the incidence rate of injury. The difference between the recurrence rate of lumbar muscle sprain and the incidence rate of sports injury is the largest, but the difference is only 19.1%. In summary, the incidence of most injury symptoms is relatively high, making it particularly important to seek suitable methods for damage repair.

**TABLE 2:** Incidence rate of sports injury and recurrence rate of sports injury.

	Incidence rate of sports injury		Recurrence rate of sports injury	
	Male	Female	Male	Female
Wrist injury	30.2%	26.6%	28.2%	20.3%
Shoulder sleeve sprain	25.3%	21.2%	16.5%	15.2%
Ankle joint sprain	24.1%	20.5%	14.1%	13.2%
Lumbar muscle sprain	23.2%	19.8%	12.7%	11.2%
Heel pain	20.2%	20.3%	16.2%	11.2%
Thigh muscle sprain	18.3%	19.2%	11.3%	12.3%
Knee joint ligament sprain	15.3%	16.5%	7.5%	10.1%
Tennis elbow	13.6%	14.8%	9.7%	6.1%
Finger sprain	14.3%	16.1%	7.2%	8.2%
Sprain of calf bone and flesh group	12.2%	11.3%	6.5%	7.5%
Eye sprain	8.6%	9.7%	4.2%	4.6%
fracture	5.1%	4.9%	3.2%	3.3%
Achilles tendon rupture	3.1%	2.3%	1.9%	1.1%

### 2.2 Investigation on repair materials for sports injury

Among the numerous symptoms of injury, sports induced bone injury is a more serious one, which is bone injury (Amanda et al., 2021). Smaller bone injuries can generally self heal, but larger bone defects are difficult to repair through self healing. In this case, it is necessary to rely on bone repair or bone replacement materials to meet the requirements (Xiyue et al., 2019). In this paper, a class of porphyrin covalent bonding based organic skeleton materials is designed to achieve precise and controllable preparation and chemical structure control of skeleton materials at the molecular level, and to prepare plastic conjugate materials.

**FIGURE 2:**
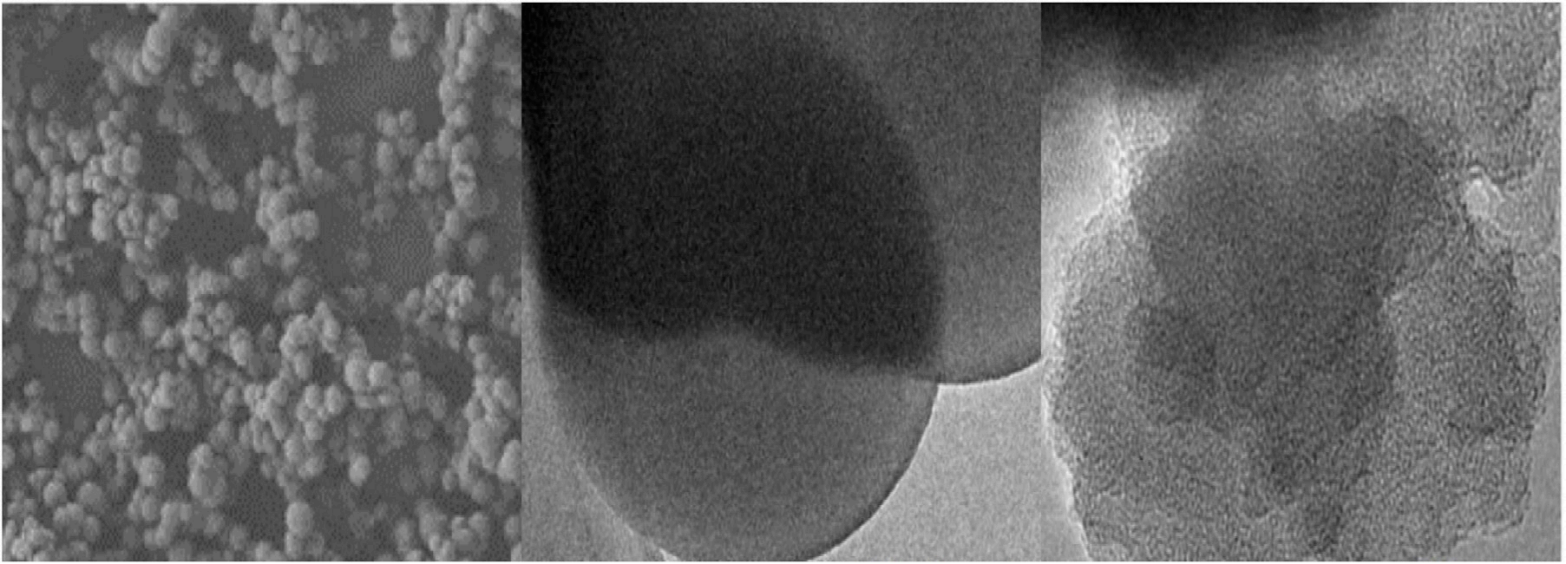
Photos of porphyrin conjugated organic skeleton materials.

Preparation of conjugated materials: This article selects pyrrole and p-phenylenediamine as substrates to prepare conjugated materials. Firstly, it is necessary to weigh 13.4 g of para benzaldehyde and add it to 200 mL of ethanol for stirring. After sufficient dissolution, this is transferred to a 500 mL flask. 6.9 mL of pyrrole, 10 mL of concentrated hydrochloric acid, and 5 mL of nitrobenzene were added to the flask, and the solution was continuously stirred to mix evenly. The flask is placed in an oil bath and kept stirring. This is maintained at 60°C, allowing the mixture to react for 12 h. The obtained product is filtered, washed with ethanol, and dried to obtain a nitrogen porphyrin conjugated organic skeleton material (Zhou, Zhuang-Ping, 2018; Lunjie et al., 2021). As shown in Figure 2, the images show scanning electron microscopy, transmission electron microscopy, and high-resolution transmission electron microscopy images of porphyrin conjugated organic skeleton materials synthesized using pyrrole and p-benzaldehyde as substrates. Secondly, PVA aqueous solution is repeatedly frozen and melted to form PVA aqueous solution to form a stable gelled state, which can form hydrogel. Finally, taking the nitrogen porphyrin conjugated organic skeleton material and hydrogel as the substrate, they are fully dissolved in organic solvent. This involves evenly mixing the acid and oxidant, and the reaction takes place at a temperature of 50°C. The reaction time is 20 h, so that a plastic conjugated material can be obtained.

Plastic conjugated material scaffold: This article uses selective laser sintering technology to mix plastic conjugated materials with biodegradable polymer-polylactide to form a plastic conjugated mixed material scaffold. Polylactide can first degrade, and then leave pores on the closed membrane. The encapsulated plastic conjugated material can be exposed to body fluids, allowing the conjugated material to exhibit its effects.

**FIGURE 3:**
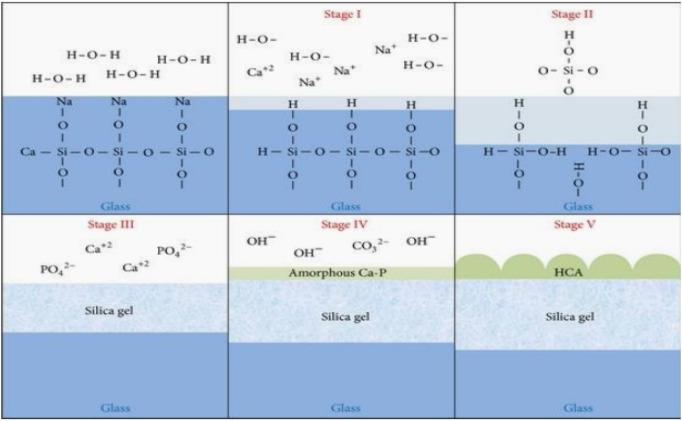
Mineralization mechanism of bone like apatite on the surface of bioglass.

Implantation and release of active ingredients: Bioactive materials can promote tissue regeneration and damage repair (Md Minhajul et al., 2020; Wei et al., 2022). $SiO_{2}-P_{2}O_{5}$ (45S5) bioglass is a bioactive material. In this paper, this material is embedded into a plastic conjugate material to form a composite material system. When it is implanted into the organism, a layer of bone like apatite structure is generated at the interface between the material and bone tissue, and a firm chemical bond can be generated at the interface between the material and bone tissue. The mineralization mechanism of 45S5 bioactive glass *in vivo* can be simply summarized as the following steps: As shown in Figure 3, a large number of cations such as ${Na}^{+}$ and ${Ca}^{2+}$ are released from the bioglass and displaced with $H^{+}$ in the solution, resulting in a large amount of $\text{Si}-{\text{OH}}^{+}$ on the glass surface: Si-O- ${Na}^{+}+H^{+}+{OH}^{-}$ →Si- ${OH}^{+}+{Na}^{+}$ + ${OH}^{-}$ . The single displacement reaction makes a large number of silicon enriched layers form on the glass surface. The increase in pH (Potential of Hydrogen) value in the solution disrupts the Si-O-Si network structure in bioglass, resulting in the formation of Si-OH (silanol) on the glass surface. The Si-OH groups on the surface of bioglass undergo a condensation reaction, forming a new silica rich sol layer on the glass surface. The ${Ca}^{2+}$ and ${{PO}_{4}}^{3-}$ ions in the solution undergo chelation reactions with Si OH, forming an amorphous $\text{CaO}-P_{2}O_{5}$ in the silicon rich layer. In the solution, ${OH}^{-}$ and ${{CO}_{3}}^{2-}$ ions form carbonate doped hydroxyapatite (HCA) with $\text{CaO}-P_{2}O_{5}$ amorphous layers on the glass surface.

Bone tissue is a complex biomaterial, and its biomechanical properties are closely related to its own chemical composition and tissue structure. In this paper, plastic conjugate materials are used to repair sports injury of bone tissue, and the mechanical properties of repair materials should be the same as that of bone tissue. The following would discuss the bearing capacity of repair materials from the mechanical perspective.

Bone is heterogeneous, anisotropic, and has visco linear elasticity properties. As a bone tissue repair material, plastic conjugated material should have similar properties. In this paper, the constitutive formula of the conjugated material anisotropic elastomer is established:

$$\delta_{\theta }=\rho_{\theta \theta }\epsilon_{\theta }$$ (1)

Generally, bone tissue repair materials are regarded as materials with transverse isotropic symmetry, and the stiffness matrix $\rho_{\theta \theta }$ can be written as follows:

$$\left|\rho_{\theta \theta }\right|=\left[\begin{array}{ccc} \rho_{11} & 0 & 0\\ 0 & \rho_{22} & 0\\ 0 & 0 & \rho_{33} \end{array}\right]$$ (2)

In the formula: $\rho_{11}$ = $\rho_{22}$ , and $\rho_{33}$ =( $\rho_{11}$ + $\rho_{22}$ )/2, so this matrix has only two different constants. This Constitutive formula can better describe the elastic properties of tissue repair materials. This formula in this article is based on the biomechanical characteristics of bone tissue repair materials under different degrees of mechanical loads to establish a mechanical model, and then obtain the constitutive formula of the repair material.

The bone tissue is mainly viscoelasticity, and the nonlinear viscoelastic constitutive model is widely used. On this basis, this paper establishes a nonlinear viscoelastic constitutive model:

$$\epsilon \left(a\right)=\int_{o}^{-a}B\left(a-A_{1}\right)\beta \left(A_{1}\right)cA_{1}+\int_{o}^{-a}\int_{o}^{-a}D\left(a-A_{1},a-A_{2}\right)\beta \left(A_{1}\right)\beta \left(A_{2}\right)dA_{1}dA_{2}+\int_{0}^{-a}\int_{0}^{-a}\int_{0}^{-a}K\left(a-A_{1},a-A_{2},a-A_{3}\right)\beta \left(A_{1}\right)\times \beta \left(A_{2}\right)\beta \left(A_{3}\right)dA_{1}dA_{2}dA_{3}$$ (3)

Early studies regarded cortical bone as an elastic body, and experimental results also demonstrated that cortical bone has anisotropy, non-uniformity, and viscoelasticity viscoplasticity. It was measured that the axial elastic modulus of cortical bone is significantly greater than the transverse elastic modulus. In the constitutive model established based on this for bone tissue repair materials, the adhesive properties make the stress-strain relationship time-dependent, with the following formula:

$$\beta \left(\epsilon ,a\right)=\beta^{0}\left(\epsilon \right)-\sum_{i=1}^{n}w\left(\epsilon ,a\right)$$ (4)

The plastic flow function γ is introduced to describe plastic strain:

$$\epsilon^{m}=ℶ\left(\frac{\partial \gamma }{\partial \gamma }\right)$$ (5)

In the formula, ℶ represents plastic rheology, and this model can accurately calculate the tensile mechanical behavior of bone tissue repair materials.

The viscoelasticity and viscoplastic properties of bone tissue repair materials determine that the stress-strain relationship is rate dependent. On this basis, a general viscoelastic-viscoplastic model can be established at low strain rate and high strain rate. The viscoelasticity and viscoplastic parts are shown as follows:

$$\beta \left(a\right)=P_{o}\epsilon^{\text{vp}}a+y_{1}\epsilon^{\text{vp}}\left(1-p^{\frac{p_{1}}{y_{1}}}\right)+y_{2}\epsilon^{\text{vp}}\left(1-p^{\frac{p_{2}}{y_{2}}}\right)$$ (6)

$$\epsilon^{vt}=\frac{\beta }{\left|\beta \right|}\left(\frac{\left|\beta \right|}{S_{o}}\right)$$ (7)

The strain of bone tissue repair materials can be divided into three parts: viscoelasticity corresponding, viscoplastic corresponding and bone reconstruction corresponding, and their strain rates are as follows:

$$\epsilon_{i}^{v}=\frac{\beta_{i}}{v_{i}}$$ (8)

$$\epsilon^{q}=ℶ\left(\frac{\partial \gamma }{\partial \gamma }\right)=ℶ*sign\left(\beta \right)$$ (9)

$$\epsilon^{l}=U*sign\left(\beta \right)$$ (10)

In the formula, $v_{i}$ is the viscosity, and U is the bone reconstruction coefficient.

The constitutive formula of bone injury is established based on the fracture characteristics of bone. From the study of the tensile yield characteristics of bone, the relationship between plastic strain and instantaneous strain of bone can be summarized, and a semi empirical formula for the yield of bone injury repair tissue under tensile force can be derived from this:

$$\beta_{i}=P_{o}\left[\epsilon_{i}-g\left(\epsilon_{i}-\epsilon_{s}\right)\right]p^{-m\left(\epsilon_{i}-\epsilon_{s}\right)}$$ (11)

In the formula, $\epsilon_{i}$ represents the instantaneous strain; $\epsilon_{s}$ is the yield strain; g is the slope of plastic strain.

Bone is a biological material, and its mechanical properties are not constant, which makes it more difficult to prepare bone tissue repair materials. The preparation of bone tissue repair materials should strive to have the same structural characteristics as bone tissue. This article applies plastic conjugated materials to sports bone injury, and should establish a true bone tissue constitutive formula to describe the mechanical adaptability of bone and reflect changes at the biological level. On this basis, the constitutive formula and mechanical property formula of plastic conjugated materials are constructed to measure the degree structure and mechanical properties of plastic conjugated materials.

## 3 Experimental testing of conjugated material properties

### 3.1 Experimental design

The demand for fitness among modern people is increasing day by day, with a considerable number of sports groups being the elderly and teenagers. However, many people, due to a lack of correct guidance, are not only unable to enhance their physical fitness, but also prone to bone injuries. Most adults are also prone to bone injury because they seldom pay attention to physical exercise in daily life, and do not do warm-up and other preparations before exercise, which also increases the demand for repair methods of sports injury. Traditional repair methods for sports injury can not meet people’s requirements. In this paper, plastic conjugated materials are used as a repair method for sports injury, Common azoporphyrins prepared using pyrrole and p-benzaldehyde as substrates are conjugated to organic frameworks through covalent conjugation. The porphyrin molecules can also be connected to organic framework materials through covalent bonds to form stable covalent bonds. Price structure to meet actual needs and the applicability of plastic conjugated materials in sports injury repair is evaluated from the degradation, compression strength, compression modulus, and the proportion of new bone formation area of plastic conjugated materials.

### 3.2 Biodegradability evaluation

Whether athletes or fitness crowd, sports injury is inevitable. The traditional injury repair materials have low biocompatibility and poor degradability, which cannot meet people’s needs. Generally speaking, after entering the human body, biomaterials would undergo hydrolysis, enzymolysis and other processes, and gradually degrade into low molecular weight compounds and other substances. The degradation products are eliminated from the body or participate in normal metabolism and disappear. However, if biodegradable biomaterials with low biodegradability enter the human body, they may remain in the human body for a long time and require secondary surgery to remove them; even the following situations may occur: undegraded biomaterials have adverse reactions with local tissues in the body, which undoubtedly increases the pain and risk of patients. This article aims to address this issue by using chemical methods to synthesize plastic conjugated tissue materials. Plastic conjugated tissue is combined with highly biodegradable polylactide to form a plastic conjugated material scaffold, in order to improve the biodegradability of biomaterials.

This experiment combines polylactide with plastic conjugated materials to form a mixed conjugated material mixture. In order to understand whether polylactide can improve the biodegradability of plastic conjugated materials and the relationship between the content of polylactide and the degradation performance of conjugated materials, mixed plastic conjugated materials with a proportion of 0, 10%, 20%, 30%, 40%, and 50% of polylactide were placed in a phosphate buffer salt solution. This is soaked at a temperature of 37°C for 28 days. There are a total of 6 groups set up in this experiment, and the group with a proportion of 0% polylactide is the control group. The other five groups were the experimental group, and the weight loss rate of the plastic conjugated material scaffold was observed every 7 days.

**FIGURE 4:**
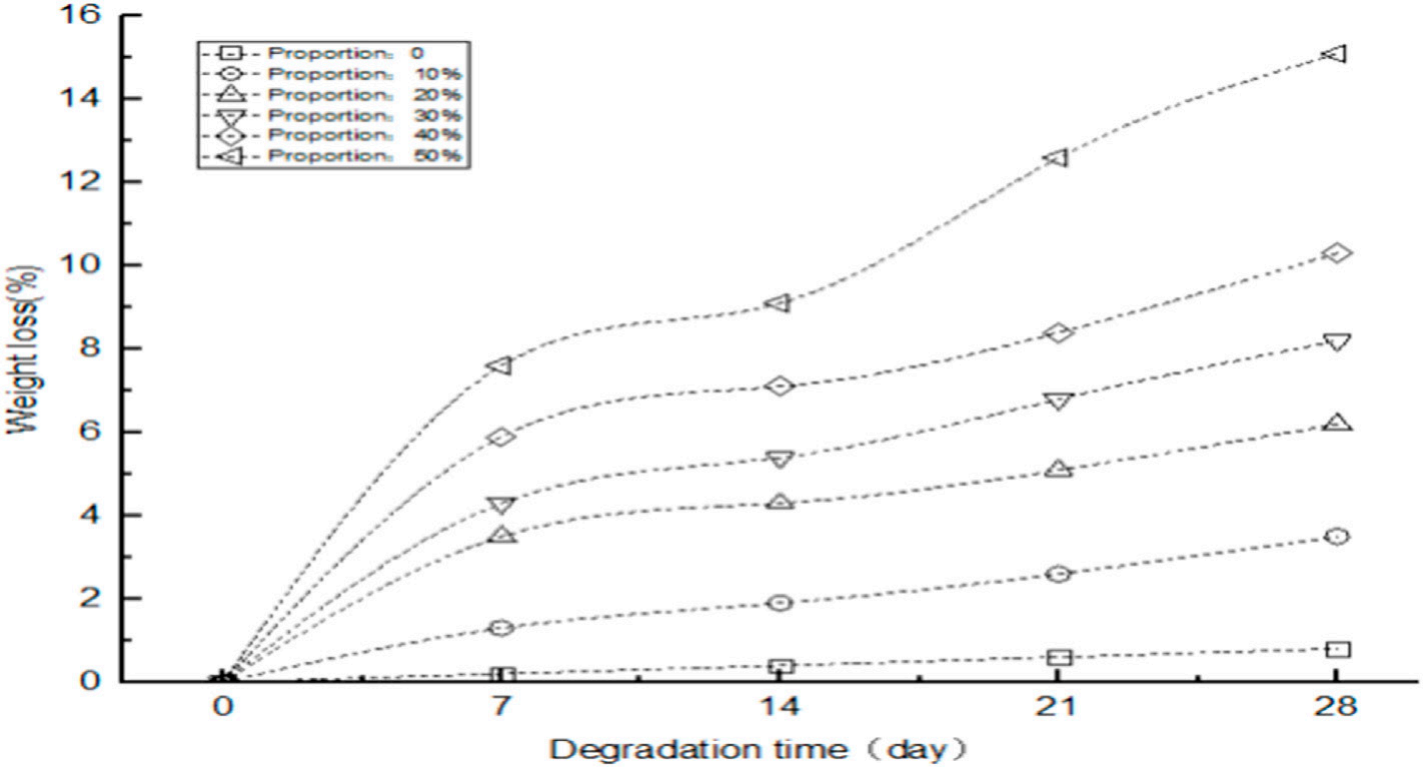
Degradation performance of plastic conjugated materials with different content of polylactide.

As shown in Figure 4, the horizontal axis represents the degradation days of the plastic conjugated material, and the vertical axis represents the variation pattern of the weight loss rate of the plastic conjugated material under different proportions of polylactide. When the content of polylactide was 0, the weight loss rate of the plastic conjugated material scaffold was very low. At the beginning, the weight loss rate was 0, and by the seventh day, the weight loss rate of the conjugated material was 0.2%. As the degradation time increased, the weight loss rate of the conjugated material slowly increased. By the 28th day, the weight loss rate of the conjugated material was only 0.8%; after adding 20% polylactide, the weight loss rate of the plastic conjugated material significantly increased. On the seventh day, the weight loss rate of the conjugated material was 1.3%, which was more than six times higher than that of the 0% polylactide; from the trend of upward extension of the line chart, within a certain range, the more the proportion of polylactide, the higher the weight loss rate of conjugated materials. When the proportion of polylactide was 50%, the weight loss rate of conjugated materials on the seventh day was 38 times that of 0% polylactide. On the 28th day, the degradation rate reached its highest value of 15.1%, which was approximately 76 times the minimum value at the beginning of the experiment. The experimental data showed that polylactide had a promoting effect on the degradation of plastic conjugated materials, greatly improving the degradability of plastic conjugated materials.

### 3.3 Compression strength evaluation

In addition to the research on the degradation rate of plastic conjugated scaffold materials, there is an important issue when implanting scaffold materials into the human body, which is whether the mechanical properties match the mechanical properties of human bones and whether they can provide a certain supporting effect for new tissues. Compared with the mechanical properties of human bones, the mechanical properties of traditional scaffold materials still have considerable shortcomings, which are prone to deformation and mechanical properties cannot play a normal supporting role. This article uses a scaffold material mixed with polylactide and plastic conjugated materials to measure and study the biomechanical properties of plastic conjugated materials.

(1) Comparison of compression strength between two methods

**FIGURE 5:**
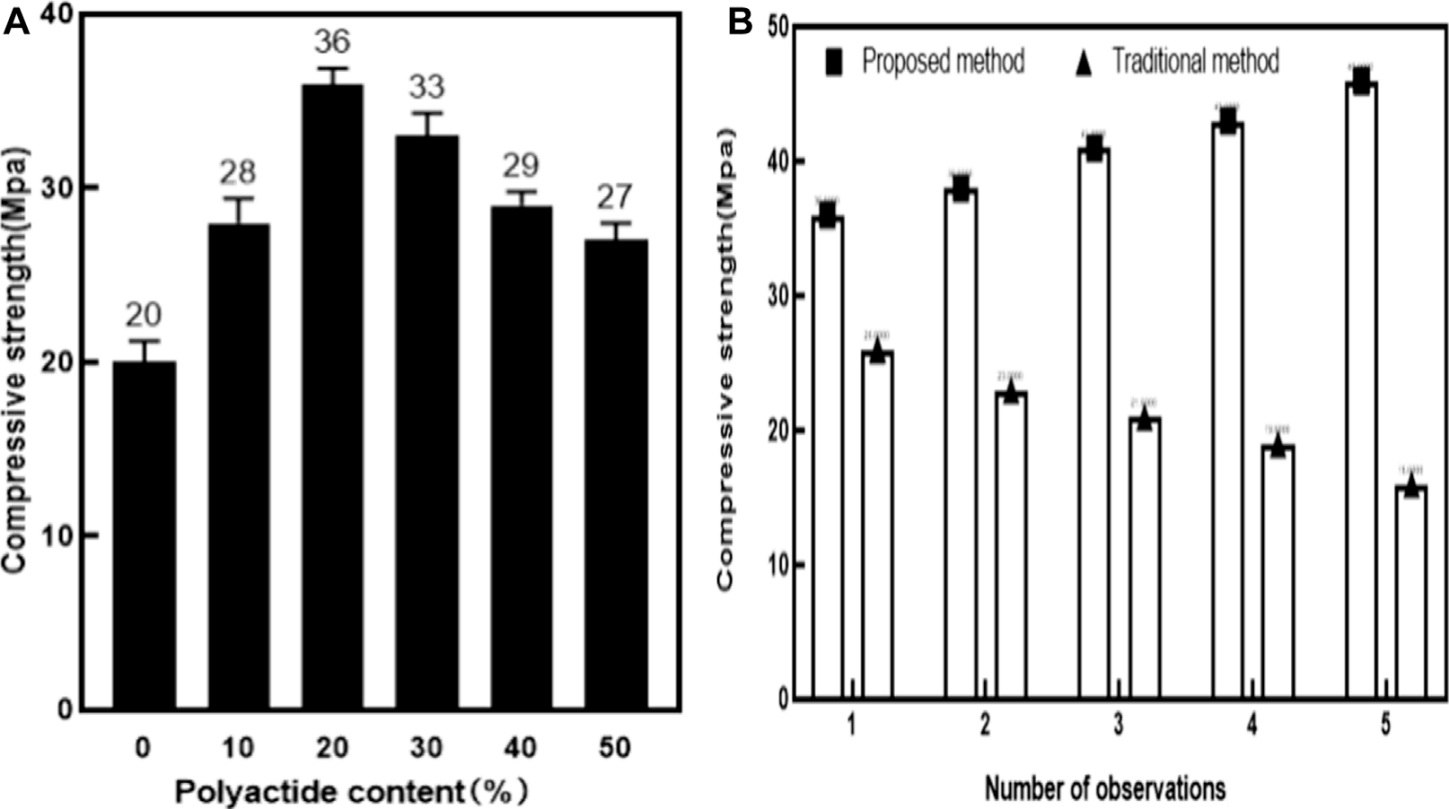
Comparison of compressive strength. Figure 5 **(A)** Comparison of compressive strength of different scaffold materials with different content of polylactide. Figure 5 **(B)** Comparison of compressive strength between two support materials.

Compression strength is one of the mechanical properties of scaffold materials. Compression strength refers to the maximum stress value that a material can withstand when subjected to compression. In order to test the ability of the scaffold material used in this article to resist deformation, multiple sets of experiments were set up to measure the compressive strength of scaffold materials with different contents of polylactide, as well as to compare the compressive strength between the method used in this article and the traditional method at (1–16) days. The experimental results are shown in Figure 5.

The experiment in Figure 5A was set up with six groups. The control group was a scaffold material with a content of 0% polylactide, while the remaining five groups were scaffold materials with a content of 10%, 20%, 30%, 40%, and 50% polylactide. Various factors such as temperature and pH value were controlled to maintain the six groups of experiments under the same conditions, and the compressive strength of each scaffold material was measured regularly.

The experiment in Figure 5B was divided into two groups: One was the control group (based on traditional methods) and the other was the experimental group (based on the method in this article). The two materials were tested under the same conditions, and the compressive strength changes of the two materials were observed and recorded on days 1, 4, 8, 12, and 16, respectively.

As shown in Figure 5A, the horizontal axis represents the proportion of polylactide content in the plastic material scaffold, while the vertical axis represents the increase in the pattern of polylactide content. Changes in compressive strength of plastic conjugated scaffold materials: As shown in Figure 5A, the compressive strength of the plastic conjugate scaffold material showed a trend of first increasing and then decreasing. Polylactide also had mechanical properties, but due to its lower mechanical strength than plastic conjugated materials, the mechanical strength of plastic conjugated scaffold materials was slightly affected as the content of polylactide increases to 30% or more. From the experimental data, it could be seen that the compressive strength of the plastic conjugated scaffold material was 20Mpa (Mega pascal) when the content of polylactide was 0. When adding 10% of polylactide to the plastic material, the compressive strength of the plastic conjugated scaffold material increased to 28Mpa. When the content of polylactide increased to 20%, the compressive strength of the plastic scaffold material reaches the highest, reaching 36Mpa, which was the peak. When the content of polylactide increased to 30%, the compressive strength of the plastic conjugated scaffold material slowly decreased, but still higher than the scaffold material with 0% of polylactide. It could be seen that the increase in polylactide helped to improve the compressive strength of plastic conjugated materials. When the content of polylactide was 30%, the compressive strength of the plastic conjugated scaffold material was the highest.

It could be seen from Figure 5B that the horizontal axis represented the number of observations during the compression strength experiment. This experiment would be observed five times. Each experiment would observe the change trend of compression strength at the same time point. The vertical axis represented the change value of compression strength of two sports injury repair materials with the increase of curing days. From Figure 5A, it could be seen that the plastic conjugate scaffold material with a 20% proportion of polylactide had the highest compressive strength value. Therefore, Figure 5B used a plastic conjugate scaffold material with a 20% proportion of polylactide and traditional bone tissue repair materials for experiments. The column chart visually presented the changes in compressive strength of two repair materials. The compressive strength of the plastic conjugate scaffold material increased with the increase of curing days. At the first observation, the compressive strength of the plastic conjugate scaffold material was 36Mpa. At the second observation, the compressive strength increased by 2Mpa. At the third and fourth observations, the compressive strength of the plastic conjugate material was 41Mpa and 43Mpa, respectively. At the fifth observation, the compressive strength had already increased by 10Mpa compared to the initial value, indicating that the plastic conjugate scaffold material was stable and safe, and its ability to resist deformation remained stable and increasing. In contrast, the compressive strength of traditional sports injury repair materials was in a downward trend. On the first day, the compressive strength of traditional damage repair materials was 26Mpa, which was reduced by 10Mpa compared to the plastic conjugate scaffold material during the same period. On the fourth day of observation, traditional damage repair materials showed a decrease of 3Mpa compared to the first day, while the difference was 15Mpa compared to the same period of plastic conjugate scaffold materials. With the number of days gradually increasing, the compressive strength of traditional sports injury repair materials was decreasing, and the gap between the compressive strength of the plastic conjugate materials and the compressive strength of the plastic conjugate materials continued to widen. On the 16th day, the compressive strength of traditional sports injury repair materials was reduced to 16Mpa, which was 10Mpa less than that on the first day. Compared with the plastic conjugate scaffold materials of the same period, this reduced by 30Mpa. It could be seen that the resistance to deformation of sports injury repair materials used in this paper was higher than that of traditional sports injury repair materials.

Table 3 lists the cumulative pore volume, total pore specific surface area, and porosity of plastic conjugated material scaffolds with different contents of polylactide. The results indicated that compared to pure plastic conjugated materials, the introduction of polylactide had to some extent improved the cumulative pore volume and porosity of plastic conjugated material scaffolds. With the increase of polylactide, the total pore specific surface area of plastic conjugated materials also showed an increasing trend within a certain range. The experimental data showed that the cumulative pore volume of the plastic conjugated material with a content of 0 in polylactide was 0.672 mL/g. As the content of polylactide continues to increase, until the polylactide content reached 20%, the cumulative pore volume reached the highest value of 0.892 mL/g. When the amount of polylactide continued to increase, the cumulative pore volume of the plastic conjugated material slowly decreased, and the cumulative pore volume of the plastic conjugated scaffold material continued to increase by 30%, 40%, and 50%. The cumulative pore volume of the plastic conjugated scaffold material was 0.856, 0.832, and 0.792, respectively.

**TABLE 3:** Pore structure parameters of plastic conjugated material scaffolds with different contents of polylactide after 7 days of curing.

Material	Accumulated pore volume (mL/g)	Total void specific surface area ( $m^{2}/$ g)	Porosity (%)
Proportion:0	0.672	69.58	40.52
Proportion:10%	0.783	72.36	44.98
Proportion:20%	0.892	73.54	46.36
Proportion:30%	0.856	70.36	45.13
Proportion:40%	0.832	69.24	44.26
Proportion:50%	0.792	68.36	43.25

From the analysis of total pore specific surface area, when the content of polylactide was 0, the total pore specific surface area of the plastic conjugated material scaffold was 69.58 $m^{2}/$ g. As the amount of polylactide increased by 10%, the total pore specific surface area also gradually increased. When the content of polylactide increased to 20%, the total pore surface area increased by 3.96 $m^{2}/$ g compared to the initial value. When increasing the amount of polylactide, the total pore specific surface area gradually decreased. When the content of polylactide was 50%, the total pore specific surface area decreased to 68.39 $m^{2}/$ g.

From the analysis of porosity, when the content of polylactide was 10% and 20%, the porosity increased by 4.46% and 1.38% compared to the initial value, respectively. When the content of polylactide was 20%, the porosity reached its peak, with a value of 44.36%. With the continuous increase of polylactide, the porosity decreased slightly. However, the porosity of the plastic material bracket with a 50% content of polylactide was still 43.25%, which was higher than the original value. From this, it could be seen that with the continuous addition of polylactide, the cumulative pore volume, total pore specific surface area, and porosity of the plastic conjugated scaffold material showed a trend of increasing first and then decreasing. The turning point occurred when the content of polylactide was 30%. However, the technology showed a downward trend in the later stage, but the decreasing data was still higher than the original values of various indicators.

### 3.4 Comparison of compression modulus between two methods

**FIGURE 6:**
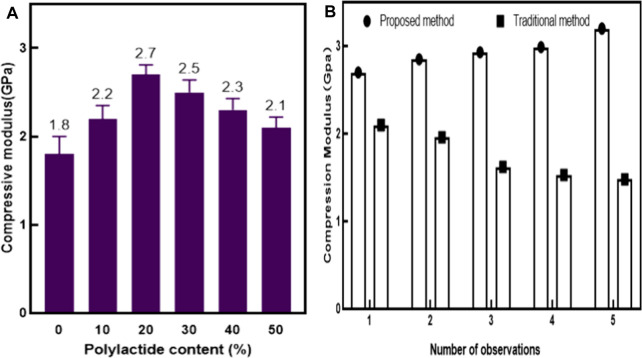
Comparison of compression modulus. Figure 6 **(A)** Comparison of compressive strength of different scaffold materials with different content of polylactide. Figure 6 **(B)** Comparison of compression modulus of two support materials.

Compression modulus is also one of the mechanical properties of scaffold materials. Compression modulus refers to the degree of elastic deformation of a material during compression, and is an important parameter of mechanical properties. The larger the compression modulus, the harder the material is and the greater its compressive strength. From Figure 6A, it could be seen that in this article, corresponding experiments were set up to detect the compressive modulus of polylactide after adding plastic conjugated materials. The content of polylactide in the plastic conjugated scaffold material in the experiment was 0, 10%, 20%, 30%, 40%, and 50%, respectively. Six sets of experiments were controlled under the same conditions and the detection data were recorded. The experimental results are shown in Figure 6.

The experiment in Figure 6B was divided into two groups: One was the control group (based on traditional methods), and the other was the experimental group (based on the method in this article). The two materials were tested under the same conditions, and the compressive modulus of the two materials was observed and recorded on days 1, 4, 8, 12, and 16, respectively.

From Figure 6A, it could be seen that the horizontal axis represented the proportion of polylactide in the plastic conjugated material, while the vertical axis represented the increase in the content of polylactide and the change in the compressive modulus of the plastic conjugated scaffold material. As shown in the figure, with the increase of the content of polylactide, the compressive modulus of the plastic conjugated scaffold material gradually increased and then decreased. When the content of polylactide was 0, the compressive modulus of the plastic conjugated scaffold material was 1.8 Gpa (Giga pascal). When the content of polylactide was 10%, the compressive modulus of the plastic conjugated scaffold material increased by 0.4 Gpa. When the content of polylactide increased to 20%, the compressive modulus of the plastic conjugated material reached its highest value, which was 2.7 Gpa. On this basis, it continued to increase the content of polyethylene glycol, and the compressive modulus gradually decreased. When the content of polyethylene glycol was 50%, the compressive modulus of the plastic conjugated scaffold material decreased to 2.1 Gpa. It could be seen that the increase of polylactide could promote the improvement of the compressive modulus of the scaffold material.

From Figure 6B, it could be seen that the horizontal axis represented the number of observations in this experiment. In order to eliminate interference from other factors, each observation was controlled at the same time point, and the vertical axis represented the changes in compressive modulus of the two repair materials at different times. It could be seen from Figure 6A that the compression modulus of the plastic conjugate scaffold material with 20% polylactide was the largest. The experiment in Figure 6B used the plastic conjugate scaffold material with 20% polylactide content and the traditional sports injury repair material for the experiment. It could be seen from the figure that, with the increase of time, the compression modulus of plastic conjugate scaffold materials kept increasing, while the compression modulus of traditional sports injury repair materials showed a downward trend. In the first test, the compressive modulus of the plastic conjugate scaffold material was 2.7 Gpa, and that of the traditional sports injury repair material was 2.1 GPa, which was 0.6 Gpa less than the plastic conjugate scaffold material. In the second observation, the compressive modulus of the plastic conjugate scaffold material increased by 5.6% compared with the first observation. Compared with the first time, the traditional sports injury repair materials decreased by 6.7%. With the increase of time, the compression modulus of plastic conjugate scaffold materials gradually increased, and the compression modulus of traditional sports injury repair materials gradually decreased. At the fifth observation, the compression modulus of plastic conjugate scaffold materials reached 3.2Gpa, which was about 2.2 times of the compression modulus of traditional sports injury repair materials. In conclusion, plastic conjugate scaffold materials had higher compression resistance than traditional sports injury repair materials.

### 3.5 Comparison of repair of radial defects and evaluation of the proportion of new bone formation area

In order to evaluate the role of plastic conjugated materials and traditional bone damage repair materials, this article set up a comparative experiment on the repair effect of radial defects. This experiment was divided into three groups: using traditional bone damage repair materials, using plastic conjugated scaffold materials with a proportion of 0% of polylactide, and using plastic conjugated scaffold materials with a proportion of 20% of polylactide for radial defect repair. The effect of radial defect repair was observed at 3 and 6 weeks after surgery.

**FIGURE 7:**
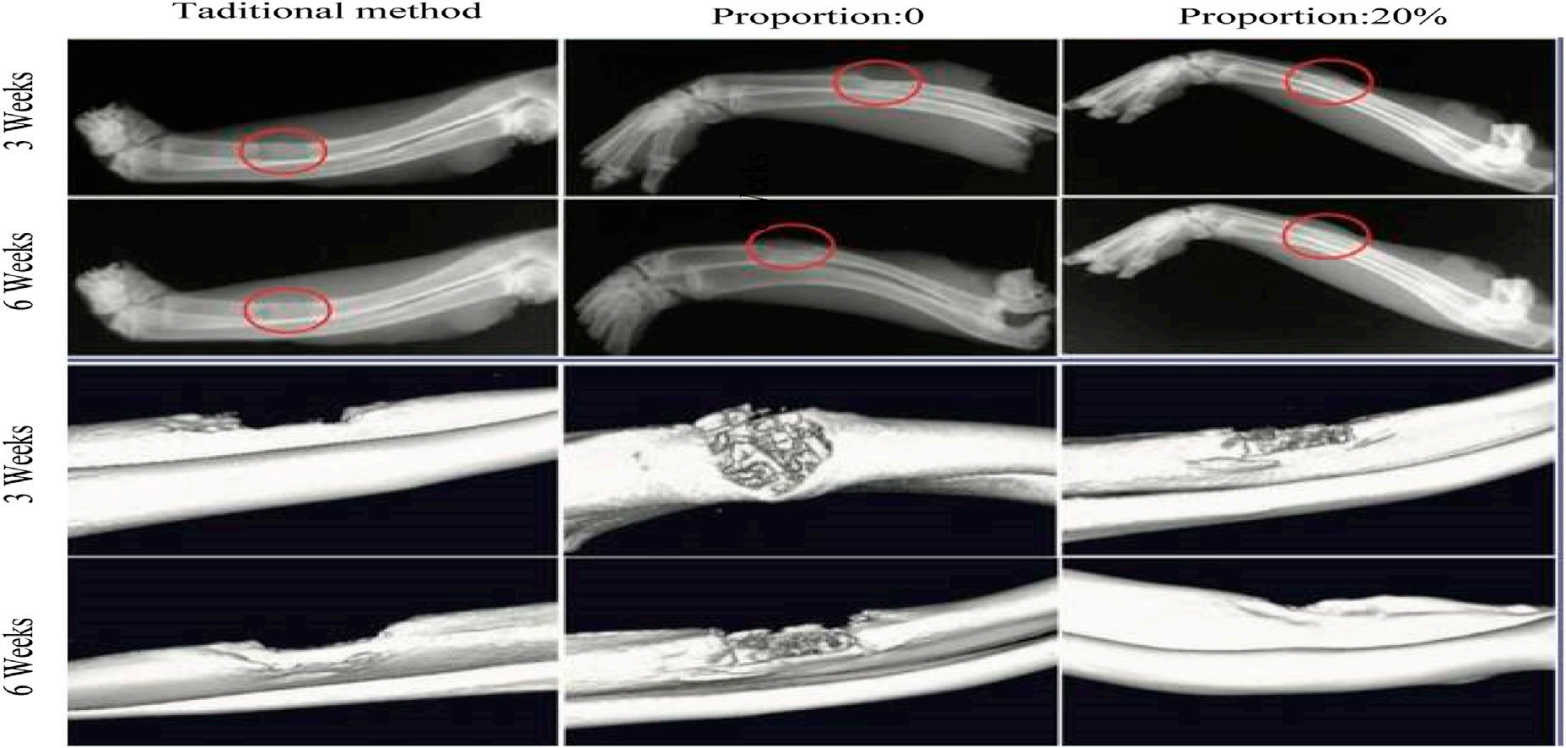
Comparison of repair effects for radial defects.

From the perspective of bone defect repair, it could be seen from Figure 7 that the red circle marked three groups of bone defect shadows. At 3 weeks after surgery, traditional methods showed more obvious bone defect shadows, while opaque calcification shadows were observed in the plastic conjugated scaffold material with a proportion of 0% and 20%, respectively. However, the bone defect shadow in the plastic conjugated scaffold material with a 0% proportion of polylactide was more pronounced than that in the plastic conjugated scaffold material with a 20% proportion of polylactide, indicating that the plastic conjugated scaffold material with a 20% proportion of polylactide had better bone formation promoting ability. At 6 weeks after surgery, the bone defect shadow of the plastic conjugated scaffold material with a 20% proportion of polylactide disappeared, and the defect area was completely connected to the host bone. The bone defect shadow was still visible using traditional methods. From the perspective of new bone formation, further observation using microcomputer tomography showed that at 3 weeks after surgery, the plastic conjugated scaffold material with a 20% proportion of polylactide showed significant new bone formation, while the plastic conjugated scaffold material with a 0% proportion of polylactide showed a small amount of new bone formation and the scaffold was clearly visible. Traditional methods generated the least amount of new bone. At 6 weeks after surgery, a plastic conjugated scaffold material with a 20% proportion of polylactide showed complete bone repair. In summary, the bone injury repair material used in this article had a shorter repair time and better repair effect than traditional methods.

In order to quantitatively analyze the repair effect of this method and traditional methods on bone, this experiment counted the new bone area of this method and traditional methods.

**FIGURE 8:**
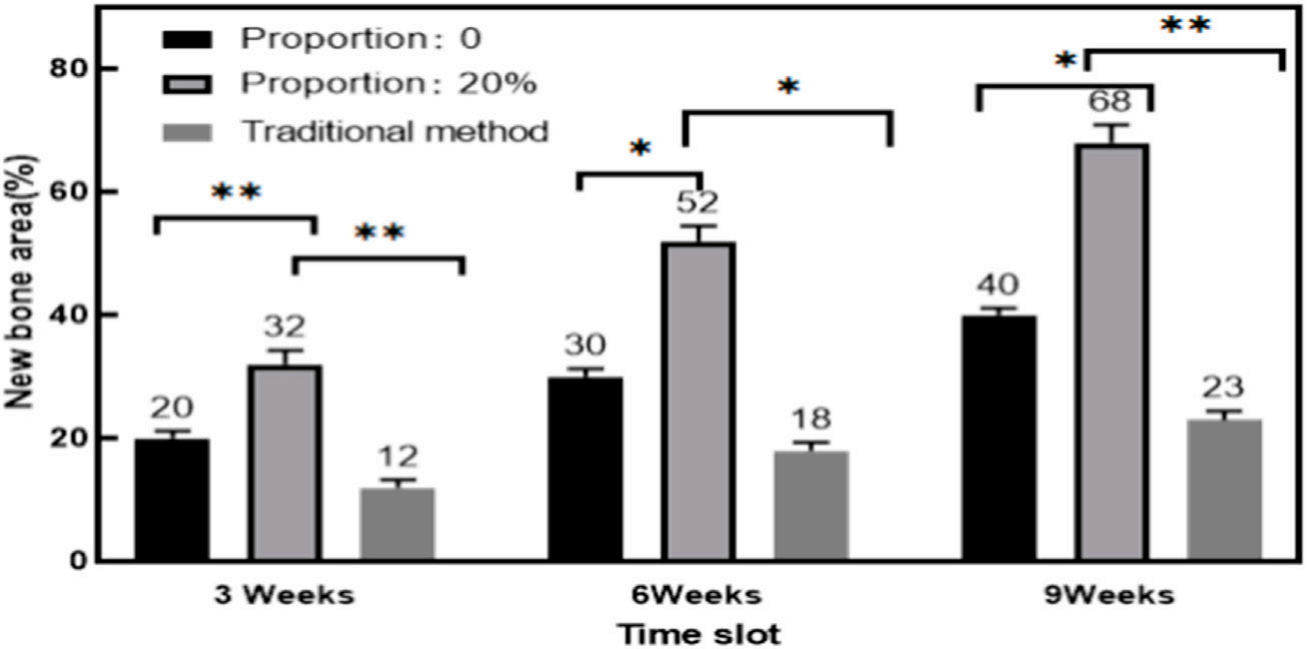
Comparison of new bone area with different repair materials.

From Figure 8, it could be seen that the horizontal axis represented the collective time period of three observations of new bone formation after surgery, and the vertical axis represented the increase in new bone area with different repair materials. The data in the figure showed that the new bone area of both the method proposed in this article and the traditional method showed a gradual increasing trend, and the plastic conjugated scaffold material with a 20% proportion of polylactide had the highest new bone area at 3, 6, and 9 weeks after surgery, which were 32%, 52%, and 68%, respectively. The traditional method of repairing materials had the smallest new bone area in three short periods of time, which were 12%, 18%, and 23%, respectively. In summary, this method had higher bone formation promoting and efficient bone reconstruction capabilities than traditional methods.

### 3.6 Analysis of chemical characteristics of azoporphyrin conjugated organic framework materials

#### 3.6.1 Elemental analysis - determine elemental composition

**FIGURE 9:**
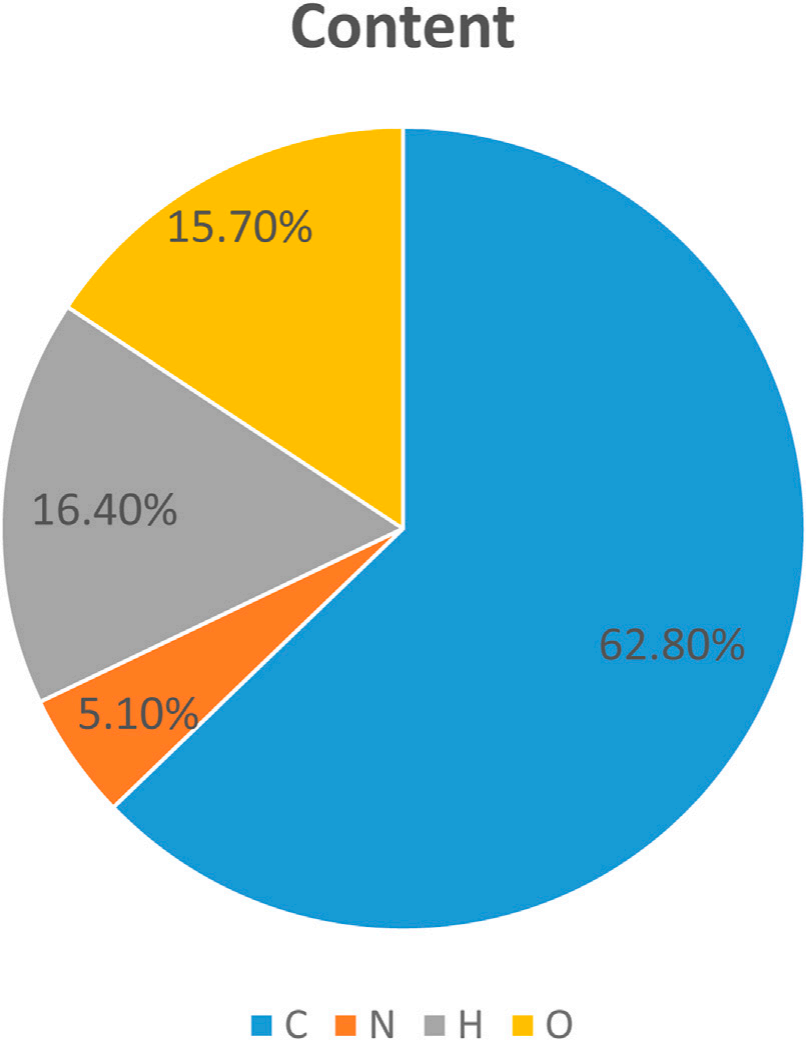
Elemental content ratio graph.

This experiment uses elemental analysis to analyze the elemental content of C, H, N, and O in azoporphyrin conjugated organic framework materials. First, prepare a sample of azoporphyrin conjugated organic framework material with a known mass, and place the sample in a heat-resistant container, then put it into a burner, and burn the sample in an oxygen or nitrogen atmosphere. During the combustion, the carbon will be converted into carbon dioxide (CO_2_), and the hydrogen will be converted into water vapor (H_2_O), and then Capture is performed through a gas collector, then gas chromatography is used to determine the content of N in the sample, the content of O is calculated using the difference method, and finally the content of each element is calculated through the known molar mass and ratio. As can be seen from Figure 9 below, C has the largest two content, reaching 62.80%. It is the main element in azoporphyrin conjugated organic framework materials, followed by H, which accounts for 16.40% in azoporphyrin conjugated organic framework materials. %, and the other two elements N and O account for 5.10% and 15.70% respectively.

#### 3.6.2 Fourier transform infrared spectroscopy (FT-IR): information that determines functional groups, chemical bonds, and molecular structure

This experiment uses Fourier transform infrared spectroscopy (FT-IR) to determine the functional groups, chemical bonds and molecular structure information in azoporphyrin conjugated organic framework materials. First, the prepared sample is placed into the FT-IR instrument for the experiment, and the sample is recorded. For the absorption or transmission of infrared light, spectral data are collected in the range of 1/4,000–1/400 cm, and then the presence of different functional groups and information related to chemical bonds are determined based on the position and intensity of the absorption peak. The experimental results show that the specifically determined functional groups are as follows: Ring structure composed of conjugated nitrogen porphyrin (4 N atoms and 4 C atoms), carbon-hydrogen bond (C-H bond), carbon-hydrogen bond (C-H bond), carbon-hydrogen bond (C-H bond), oxygen-hydrogen bond (O-H bond), carbon-oxygen bond (C-O bond), nitrogen-oxygen bond (N-O bond) and other functional groups.

## 4 Conclusion

The research and development of bone tissue injury treatment and functional recovery methods in sports has always been an important issue that needs to be solved in the development process of modern sports. It is expected that biomaterials used to repair sports bone tissue injury have the characteristics of good histocompatibility with human body, easy tissue absorption, non infection, non adhesion and fracture, and non rejection. The plastic conjugated materials used in this article highlighted significant advantages. With the increase of polylactide, the degradation performance of conjugated materials gradually improved, eliminating concerns about the long-term retention of repair materials in the human body. Secondly, compared with traditional methods, the compression strength and compression modulus of this method were higher than those of traditional sports injury repair materials, indicating that the mechanical properties of plastic conjugated strength of materials were stronger than those of traditional methods, and the repair time and repair effect of bone defects were also better than those of traditional repair materials. Finally, the generation of new bone area between the materials used in this article and traditional repair materials was tested, and it was found that plastic conjugated materials had a significant promoting effect on the formation of new bone. The shortcomings of this article were that the data collection was not comprehensive enough and the research method was single. In future studies, the number of samples would be increased. The experimental design would be improved, and more refined and comprehensive data analysis methods would be adopted.

## Data Availability

The original contributions presented in the study are included in the article/Supplementary Material, further inquiries can be directed to the corresponding author.
